# Role of Preoperative Administration of Vitamin D and Calcium in Postoperative Transient Hypocalcemia after Total Thyroidectomy

**DOI:** 10.7759/cureus.4579

**Published:** 2019-04-30

**Authors:** Muhammad Z Malik, Ahsan A Mirza, Sarosh Afzal Farooqi, Noman A Chaudhary, Mahnoor Waqar, Hamza Waqar Bhatti

**Affiliations:** 1 Surgery, Pakistan Atomic Energy Commission General Hospital, Islamabad, PAK; 2 Surgery, Holy Family Hospital, Rawalpindi, PAK; 3 Surgery, Rawalpindi Medical University, Rawalpindi, PAK

**Keywords:** hypocalcemia, thyroidectomy, calcium, vitamin d

## Abstract

Introduction

Thyroid surgery is one of the most frequently performed surgical procedures worldwide. Total thyroidectomy is a recommended procedure for most of the thyroid diseases. The most common complication resulting after this surgery is transient hypocalcemia - the incidence is 24% - which increases the morbidity rate and increases the length of stay in the hospital. The objective of our study was to compare the frequency of transient hypocalcemia after vitamin D and calcium supplementation with the control group for patients undergoing total thyroidectomy.

Patients and methods

It was a randomized controlled trial conducted at Department of Surgery, Pakistan Atomic Energy Commission General Hospital, Islamabad, Pakistan from January 2017 to July 2017. A total of 92 patients of both genders undergoing total thyroidectomy were included in the study. Patients undergoing reoperation for thyroid disease, American Society of Anesthesiologists (ASA) grade 3 or above, patients with chronic renal failure, preoperative hypocalcemia or hypercalcemia were excluded. The patients were sorted into two groups by lottery method; Group 1 in which Vitamin D (2,00,000 IU) and calcium (1 gm) was given 24 hours preoperatively. Group 2 was the control group. Total thyroidectomy was done and serum calcium levels were evaluated immediately after surgery on day two, seven and on the 30th day. The final outcome was measured at one month. Data was analyzed via the Statistical Package for Social Sciences version 22.0 (IBM Corp, Armonk, NY, USA). P value ≤ 0.05 was considered significant.

Results

The age ranged from 18 to 65 years with the mean age of 38.673 ± 8.63 years in group 1 while 41.217 ± 9.52 years in group 2, mean preoperative calcium level was 9.482 ± 0.49 mg/dl in group 1 and 9.678 ± 0.54 mg/dl in group 2. Hypocalcemia was seen in 3 (6.5%) in group 1 as compared to 12 (26.1%) patients in group 2 (p = 0.011).

Conclusion

Preoperative oral calcium and vitamin D supplements may prevent postoperative hypocalcemia, allowing a safe and early discharge. This will ultimately lead to improved patient satisfaction and significant cost savings.

## Introduction

Thyroid surgery is one of the most frequently performed surgical procedures worldwide [1]. Nowadays, total thyroidectomy is the recommended procedure for thyroid disease [2]. As total thyroidectomy is the procedure of choice, the most common complication resulting after this surgery is transient hypocalcemia - the incidence being 24% - which increases the morbidity rate and increases the length of hospitalization [3-7]. Other complications of thyroidectomy include recurrent laryngeal nerve injury which leads to hoarseness of voice, postoperative hemorrhage, dysphagia due to inflammation of the tissues surrounding the esophagus, seroma formation, Horner's syndrome due to injury to the cervical sympathetic chain, and poor healing of the wound with hypertrophy of the scar or wound infection [8,9].

Advances in surgical techniques have evolved to preserve the parathyroid gland function, which helps to prevent permanent hypocalcemia and its incidence now has reduced to 1-2% [3-5]. However, transient hypoparathyroidism still occurs resulting in transient hypocalcemia. It occurs due to the age, parathyroid gland handling, devascularization, venous congestion, post-surgical local site edema and neck dissection [5,6,10]. Prescribing preoperative vitamin D and calcium decreases the incidence of transient hypocalcemia after total thyroidectomy from 25.9% to 6.8% as compared to the control group [10].

Hypocalcemia after total thyroidectomy is usually transient but it is of main concern as it requires either prolonged stay in the hospital or readmission [6]. During the first 24 hours bleeding is the main complication, but from the second day to six months, transient hypocalcemia is of main concern [4]. Hypocalcemia can be evaluated symptomatically as well as from laboratory testing. Signs and symptoms of hypocalcemia include numbness, tingling, and carpopedal spasm [5]. Preoperative and postoperative administration of oral calcium along with vitamin D prevents postoperative transient hypocalcemia after thyroidectomy [10-12].

Total thyroidectomy is the procedure of choice in our population with the preservation of the parathyroid gland. As transient hypocalcemia is common in post-thyroidectomy patients and increases the morbidity rate, giving vitamin D and calcium preoperatively can reduce the burden of postoperative transient hypocalcemia and it will be helpful in decreasing the morbidity rate due to post-thyroidectomy transient hypocalcemia. The objective of our study is to compare the frequency of transient hypocalcemia after vitamin D and calcium supplementation with the control group for patients undergoing total thyroidectomy.

## Materials and methods

It was a randomized controlled trial conducted at the Department of Surgery, Pakistan Atomic Energy Commission General Hospital, Islamabad, Pakistan from January 2017 to July 2017. A total of 92 patients of both genders - between the age of 18-65 years and undergoing total thyroidectomy - were included in the study. Patients undergoing reoperation for thyroid disease, American Society of Anesthesiologists (ASA) grade 3 or above, patients with chronic renal failure, preoperative hypocalcemia or hypercalcemia were excluded from the study. Patients gave written informed consent before participating in the study. Once the consent was obtained, assessments of the trial outcome measures using standard assessment forms were undertaken. After the baseline measurements were carried out, an administrative assistant assigned a numerical registration number to the patients by using a lottery method. Patients then were randomly allocated into two groups; Group 1 (n = 46) in which Vitamin D (2,00,000 IU) and calcium (1 gm) was given 24 hours pre-operatively. Group 2 (n = 46) was the control group. Patients underwent a standardized subjective and objective examination, as recommended. A thorough pre-anesthetic evaluation was done and necessary investigations such as hemoglobin, blood group and Rhesus (Rh) typing, urine for albumin, sugar, bleeding time, clotting time were obtained.

Total thyroidectomy was done and serum calcium levels were evaluated. Total thyroidectomy was defined as complete removal of the thyroid gland with preservation of the parathyroid gland. Hypocalcemia was defined as a decrease in serum calcium level less than 8.5 mg/dl after thyroid surgery within one month. The collected data was entered and analyzed with the computer software, Statistical Package for Social Sciences (SPSS) version 20.0 (IBM Corp, Armonk, NY, USA). Qualitative variables like gender, hypocalcemia and ASA score were measured as frequencies and percentages. For quantitative variables like age and calcium level, mean ± standard deviation (SD) was calculated. The chi-square test was applied to compare the frequency of hypocalcemia between the two groups. Effect modifiers like age, gender, ASA score, and the reason for thyroidectomy were controlled by stratification, the chi-square test was applied, p-value ≤ 0.05 was considered significant.

## Results

The age ranged from 18 to 65 years with a mean age of 38.673 ± 8.63 years in group 1 while 41.217 ± 9.52 years in group 2, mean pre-operative calcium levels were 9.482 ± 0.49 mg/dl in group 1 and 9.678 ± 0.54 mg/dl in group 2 (Table 1).

**Table 1:** Mean age and preoperative calcium levels in group 1 and group 2.

Variables	Group 1 (Mean ± SD)	Group 2 (Mean ± SD)
Age (years)	38.673 ± 8.63	41.217 ± 9.52
Preoperative calcium (mg/dl)	9.482 ± 0.49	9.678 ± 0.54

Male and female distribution in group 1 was 22 (47.8%) and 24 (52.2%) respectively, while in group 2 was 27 (58.7%) and 19 (41.3%), respectively. In group 1 patients having ASA grade 1 were 37 (80.7%) and patients with ASA grade 2 were nine (19.6%), while in group 2 the number of patients with ASA grade 1 was 23 (50%) and ASA grade 2 was 23 (50%). Gender and ASA grade distribution between both groups is shown in Table 2.

**Table 2:** Distribution of gender and ASA grades among group 1 and group 2. ASA: American Society of Anesthesiologists

Variables	Groups	Group 1 n (%)	Group 2 n (%)
Gender	Male	22 (47.8%)	27 (58.7%)
	Female	24 (52.2%)	19 (41.3%)
ASA	Grade 1	37 (80.7%)	23 (50%)
	Grade 2	9 (19.6%)	23 (50%)

Hypocalcemia after total thyroidectomy was seen in three (6.5%) patients in group 1 as compared to 12 (26.1%) in group 2. There was a statistically significant difference among those who developed hypocalcemia in groups 1 & 2 (P = 0.011) as shown in Table 3.

**Table 3:** Frequency distribution of postoperative hypocalcemia after total thyroidectomy. *p-value ≤ 0.05 was considered significant.

Hypocalcemia	Group 1 n (%)	Group 2 n (%)	Total (n = 92)	p-value*
Yes	3 (6.5%)	12 (26.1%)	15	0.011
No	43 (93.5%)	34 (73.9%)	77	

Stratification of hypocalcemia with respect to age, gender, ASA score and reason of thyroidectomy in group 1 and group 2 is shown in Table 4.

**Table 4:** Stratification of postoperative hypocalcemia with respect to age, gender, ASA score and reason of thyroidectomy. ASA: American Society of Anesthesiologists.

Variable	Groups	Group 1 (n = 46)		Group 2 (n = 46)		p-value
		Hypocalcemia (n = 3)	Normocalcemia (n = 12)	Hypocalcemia (n = 12)	Normocalcemia (n = 34)	
Age	18-40	1	28	6	17	0.017
	41-65	2	15	6	17	0.026
Gender	Male	3	19	8	19	0.017
	Female	0	19	4	15	0.018
ASA	Grade 1	2	35	5	18	0.055
	Grade 2	1	8	7	16	0.256
Reason of thyroidectomy	Thyroid cancer	2	1	1	3	0.270
	Toxic thyroid nodule	0	5	3	4	0.091
	Multinodular goiter	0	13	2	15	0.664
	Graves’ disease	0	8	4	10	0.094
	Thyroid nodule	1	8	2	2	0.028

## Discussion

After thyroidectomy postoperative hypocalcemia is a common complication. Hypocalcemia due to hypoparathyroidism may present clinically with peri-oral and peripheral paresthesias, muscle spasms, carpopedal spasm or tetany, and/or confusion. According to a study conducted by Baldassarre et al., the incidence of hypocalcemia after thyroidectomy was 5.5% [5]. Our study shows that the combination of preoperative and postoperative oral calcium and vitamin D supplements can reduce the incidence of hypocalcemia after total thyroidectomy. In fact, this treatment avoided a significant decrease in serum calcium levels. Hypocalcemia was seen in three patients (6.5%) in group 1 as compared to 12 patients (26.1%) in group 2. However, hypocalcemia was due to the accidental removal of parathyroid tissue, as confirmed by histological analysis. The implementation of oral calcium and vitamin D is not new. However, this protocol is usually applied only in the sudden postoperative period. The role of routine calcium supplements in the prevention of hypocalcemia after thyroidectomy was evaluated in two recent studies. According to a study conducted by Bellantone et al., only three out of 26 patients (11%) receiving an oral calcium supplement developed hypocalcemia after total thyroidectomy whereas, 11 out of 27 patients (40%), not receiving a calcium supplement developed the same [13]. These studies suggest that the administration of calcium supplements prevents the development of hypocalcemia after thyroid surgery. Bellantone et al. also demonstrated that the combination of vitamin D and oral calcium supplements resulted in a significantly higher serum calcium concentrations on the second and the third postoperative days thus, decreasing the incidence of hypocalcemia [13]. Although some studies report that vitamin D administration inhibits the secretion of intact parathyroid hormone (iPTH) by normally functioning parathyroid glands yet, others claim that iPTH secretion is not affected by vitamin D administration in the patients after thyroid surgery [12,14]. Therefore, a combination of vitamin D and calcium supplements can be administered to the patients undergoing total thyroidectomy. The dosages and duration of calcium and vitamin D administration are also of concern. A higher level of serum calcium was measured in the patients who received vitamin D and calcium supplements before undergoing thyroid surgery and only a few of them developed hypocalcemia [12]. Many biochemical markers are being used to predict the development of hypocalcemia after thyroid surgery to reduce the length of the hospital stay [14]. Although serum calcium assay has been used to predict the development of hypocalcemia after the thyroid surgery; however, due to postoperative hemodilution, the measurement of total serum calcium may yield inaccurate results [15,16]. Extensive research has been done on the value of the parathyroid hormone (PTH) in predicting post-thyroidectomy hypocalcemia [17-20]. Although postoperative evaluation of iPTH may decrease the length of the hospitalization, rapid access to results of postoperative PTH measurement is not widely available in many hospitals. In addition, there is no consensus on the threshold of iPTH and the optimal timing for its measurement after thyroidectomy [21]. The measurement of iPTH on the first day after thyroidectomy has been shown to be useful in predicting the development of post-thyroidectomy hypocalcemia [22]. The serum concentration of iPTH after one to six hours following thyroidectomy has demonstrated a higher accuracy in predicting hypocalcemia. However, it is not 100% accurate [22]. Both absolute and relative decrease in the level of PTH has been used to predict hypocalcemia with similar accuracy and, although both levels are similar, they may vary between institutions [21-24]. The prevention of postoperative hypocalcemia will lead to a significant decrease in the length of stay in the hospital.

Several strategies have been adopted for conserving calcium level after thyroidectomy. To decrease the symptoms of hypocalcemia, some surgeons prefer to prescribe oral calcium, whereas others prefer to treat patients with postoperative hypocalcemia. They routinely check hypocalcemia symptoms in the postoperative period and measure serum calcium. Also, they educate the patients on the symptoms of hypocalcemia before discharging them from the hospital. Roh and Park demonstrated that administration of oral calcium and vitamin D reduces the incidence and severity of hypocalcemia after total thyroidectomy [15]. Moore showed that oral calcium administration may lead to an earlier discharge on the second postoperative day without the development of hypocalcemia [25]. Sanabria et al. evaluated four randomized clinical trials in a meta-analysis. They found that the administration of calcium and vitamin D supplements decreases the incidence of hypocalcemia symptoms after thyroidectomy [26]. The main preventive measure is preserving parathyroid glands by careful dissection during the surgery; also parathyroid autotransplantation has been shown to prevent permanent hyperparathyroidism after total, subtotal, or completion thyroidectomy for benign or malignant diseases of the thyroid gland. It has been reported that patients who received parathyroid autotransplantation had a significantly lower risk of permanent hyperparathyroidism once postoperative hypocalcemia occurred [27,28]. Abboud et al. conducted a retrospective study involving 252 patients who underwent total thyroidectomy. They found that routine autotransplantation of at least one parathyroid gland along with routine calcium and vitamin D supplementation during total thyroidectomy efficiently reduced symptomatic hypocalcemia and permanent hyperparathyroidism [29].

## Conclusions

Our study shows that postoperative hypocalcemia develops in all age groups in both males and females who undergo total thyroidectomy without preoperative calcium and vitamin D supplements. Preoperative oral calcium and vitamin D supplements may prevent postoperative hypocalcemia, allowing a safe and early discharge from the hospital. This will ultimately lead to improved patient satisfaction and significant cost savings. This will also lead to a decreased length of hospital stay as postoperative hypocalcemia lengthens the stay in the hospital.
